# [Hydridotris(pyrazol-1-yl-κ*N* ^2^)borato]bis­(methyl­amino-κ*N*)(triphenyl­phos­phine-κ*P*)ruthenium(II) chloride dichloro­methane solvate monohydrate

**DOI:** 10.1107/S1600536812042110

**Published:** 2012-10-13

**Authors:** Yi-Dong Lin, Chin-Pei Chang, Quan-Sheng Huang, Yih-Hsing Lo

**Affiliations:** a Department of Applied Physics and Chemistry, Taipei Municipal University of Education, Taipei 10048, Taiwan

## Abstract

The title salt, [Ru(Tp)(CH_5_N)_2_(PPh_3_)]Cl·CH_2_Cl_2_·H_2_O [where Tp is (C_3_H_3_N_2_)_3_BH and PPH_3_ is C_18_H_15_P], has the Ru^III^ atom in an octa­hedral geometry; one of the Ru—N(Tp) bonds [2.135 (8) Å] is slightly longer than another two, owing to the *trans* influence of PPh_3_ ligand. N—H⋯Cl and O—H⋯Cl hydrogen bonding leads to the formation of layers parallel to (100).

## Related literature

For general background, see: Alcock *et al.* (1992 ▶); Burrows *et al.* (2001 ▶); Pavlik *et al.* (2005 ▶); Slugovc *et al.* (1998 ▶).
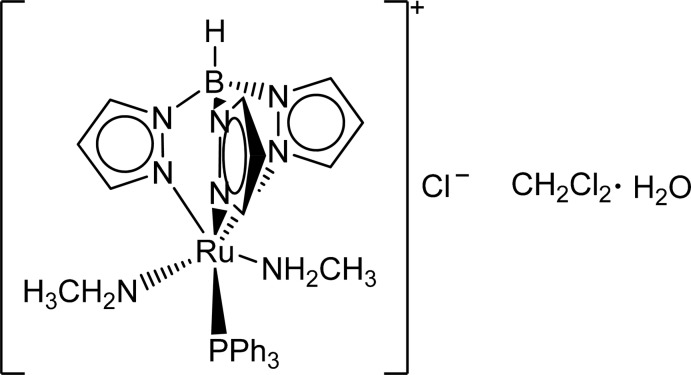


## Experimental

### 

#### Crystal data

[Ru(C_9_H_10_BN_6_)(CH_5_N)_2_(C_18_H_15_P)]Cl·CH_2_Cl_2_·H_2_O
*M* *_r_* = 776.89
Monoclinic, 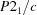

*a* = 12.3791 (8) Å
*b* = 13.1285 (9) Å
*c* = 21.5723 (15) Å
β = 97.405 (4)°
*V* = 3476.7 (4) Å^3^
*Z* = 4
Mo *K*α radiation
μ = 0.77 mm^−1^
*T* = 200 K
0.25 × 0.13 × 0.06 mm

#### Data collection

Nonius KappaCCD diffractometer
Absorption correction: multi-scan (Blessing, 1995 ▶) *T*_min_ = 0.832, *T*_max_ = 0.956
22188 measured reflections
6102 independent reflections
2862 reflections with *I* > 2σ(*I*)
*R*_int_ = 0.110

#### Refinement

*R*[*F* ^2^ > 2σ(*F* ^2^)] = 0.071
*wR*(*F* ^2^) = 0.167
*S* = 0.99
6102 reflections
408 parameters
H-atom parameters constrained
Δρ_max_ = 1.31 e Å^−3^
Δρ_min_ = −0.82 e Å^−3^

### 

Data collection: *COLLECT* (Nonius, 2000 ▶); cell refinement: *HKL* *SCALEPACK* (Otwinowski & Minor, 1997 ▶); data reduction: *HKL* *DENZO* (Otwinowski & Minor, 1997) ▶ and *SCALEPACK*; program(s) used to solve structure: *SHELXS97* (Sheldrick, 2008 ▶); program(s) used to refine structure: *SHELXL97* (Sheldrick, 2008 ▶); molecular graphics: *ORTEP-3 for Windows* (Farrugia, 1997 ▶); software used to prepare material for publication: *WinGX* (Farrugia, 1999 ▶).

## Supplementary Material

Crystal structure: contains datablock(s) I, global. DOI: 10.1107/S1600536812042110/ng5297sup1.cif

Structure factors: contains datablock(s) I. DOI: 10.1107/S1600536812042110/ng5297Isup2.hkl

Additional supplementary materials:  crystallographic information; 3D view; checkCIF report

## Figures and Tables

**Table 1:** Hydrogen-bond geometry (Å, °)

*D*—H⋯*A*	*D*—H	H⋯*A*	*D*⋯*A*	*D*—H⋯*A*
N7—H7*B*⋯Cl3	0.92	2.54	3.430 (7)	164
N8—H8*A*⋯Cl3	0.92	2.37	3.293 (6)	178
O1—H1*A*⋯Cl3	0.83	2.58	3.342 (7)	154
O1—H1*B*⋯Cl3^i^	0.83	2.32	3.136 (7)	166

Symmetry code: (i) 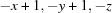.

**Fig. 1.**
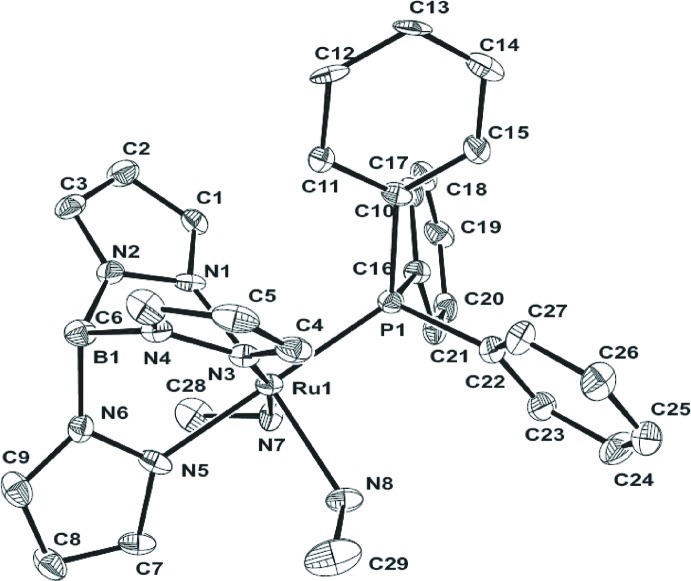
Molecular structure of the title compound with labelling and displacement ellipsoids drawn at the 30% probability level (H atoms are shown as spheres of arbitrary radius).

## supplementary crystallographic information

### Comment

Ruthenium(II) hydridotripyrazolylborate complexes, Ru(Tp), are of interest for stoichiometric and catalytic transformations of organic molecules (Pavlik *et al.*, 2005). The complex RuCl(Tp)(PPh_3_)_2_ (Alcock *et al.*, 1992) has been used as the starting material for the synthesis of several complexes because of its substitutionally labile chloride and phosphines (Burrows, 2001). The development of Tp chemistry within group VIII has picked up the pace since then.

Treatment of the complex [Ru(Tp)(PPh_3_)_2_Cl] reacted with methyl amine in warm methanol affording the title compound [Ru(NH_2_CH_3_)_2_(Tp)(PPh_3_)]Cl.CH_2_Cl_2_.H_2_O (Fig. 1). The ν(B—H) vibration of the title complex is found at 2481 cm^-1^, which is characteristic of Tp bound to a metal center in a terdentate (N,*N*,*N*) manner. Yellow crystals were obtained by slow diffusion of hexane into a CHCl_3_ solution at room temperature for 3 d. The coordination geometry is approximately octahedral and the bite angle of the Tp ligand produces an average produces an average N—Ru—N angle of 86.07° only slightly distorted from 90°. One of the Ru—N(Tp) bond length (2.135 (8) Å) is slightly longer than another two due to the *trans* influence of PPh_3_ ligand (Slugovc *et al.* 1998).

### Experimental

The synthesis of the title compound was carried out as follows. To a solution of [(Tp)(PPh_3_)_2_RuCl] (0.39 g, 0.45 mmol) in methanol (20 ml), an excess of methyl amine were added. The mixture was heated using a warm water bath for 30 min. A deep yellow color developed during this time. The reaction mixture was stirred for a further 6 h at room temperature (298 K). Then it was concentrated to approximately half of the volume and cooled to 273 K. The yellow precipitate was filtered off, washed with ethanol and ether and dried was dried under vacuum to give the title compound.

### Refinement

The H atoms were placed in idealized positions and constrained to ride on their parent atoms, by C—H = 0.95 and 0.98 Å with *U*_iso_(H) = 1.2*U*_eq_(C) and 1.5*U*_eq_(C), N—H = 0.92 Å with *U*_iso_(H) = 1.2*U*_eq_(N), and B—H = 1.0 Å with *U*_iso_(H) = 1.2*U*_eq_(B).

### Crystal data

**Table:**

[Ru(C_9_H_10_BN_6_)(CH_5_N)_2_(C_18_H_15_P)]Cl·CH_2_Cl_2_·H_2_O	*F*(000) = 1592
*M**_r_* = 776.89	*D*_x_ = 1.484 Mg m^−^^3^
Monoclinic, *P*2_1_/*c*	Mo *K*α radiation, λ = 0.71073 Å
Hall symbol: -P 2ybc	Cell parameters from 973 reflections
*a* = 12.3791 (8) Å	θ = 1.7–25.0°
*b* = 13.1285 (9) Å	µ = 0.77 mm^−^^1^
*c* = 21.5723 (15) Å	*T* = 200 K
β = 97.405 (4)°	Prism, pale yellow
*V* = 3476.7 (4) Å^3^	0.25 × 0.13 × 0.06 mm
*Z* = 4	

### Data collection

**Table:**

Nonius KappaCCD diffractometer	6102 independent reflections
Radiation source: fine-focus sealed tube	2862 reflections with *I* > 2σ(*I*)
Graphite monochromator	*R*_int_ = 0.110
φ and ω scans	θ_max_ = 25.0°, θ_min_ = 1.7°
Absorption correction: multi-scan (Blessing; 1995)	*h* = −14→14
*T*_min_ = 0.832, *T*_max_ = 0.956	*k* = −13→15
22188 measured reflections	*l* = −25→25

### Refinement

**Table:**

Refinement on *F*^2^	Primary atom site location: structure-invariant direct methods
Least-squares matrix: full	Secondary atom site location: difference Fourier map
*R*[*F*^2^ > 2σ(*F*^2^)] = 0.071	Hydrogen site location: inferred from neighbouring sites
*wR*(*F*^2^) = 0.167	H-atom parameters constrained
*S* = 0.99	*w* = 1/[σ^2^(*F*_o_^2^) + (0.0634*P*)^2^] where *P* = (*F*_o_^2^ + 2*F*_c_^2^)/3
6102 reflections	(Δ/σ)_max_ = 0.001
408 parameters	Δρ_max_ = 1.31 e Å^−^^3^
0 restraints	Δρ_min_ = −0.82 e Å^−^^3^

### Special details

**Table:**

Geometry. All e.s.d.'s (except the e.s.d. in the dihedral angle between two l.s. planes) are estimated using the full covariance matrix. The cell e.s.d.'s are taken into account individually in the estimation of e.s.d.'s in distances, angles and torsion angles; correlations between e.s.d.'s in cell parameters are only used when they are defined by crystal symmetry. An approximate (isotropic) treatment of cell e.s.d.'s is used for estimating e.s.d.'s involving l.s. planes.
Refinement. Refinement of *F*^2^ against ALL reflections. The weighted *R*-factor *wR* and goodness of fit *S* are based on *F*^2^, conventional *R*-factors *R* are based on *F*, with *F* set to zero for negative *F*^2^. The threshold expression of *F*^2^ > σ(*F*^2^) is used only for calculating *R*-factors(gt) *etc*. and is not relevant to the choice of reflections for refinement. *R*-factors based on *F*^2^ are statistically about twice as large as those based on *F*, and *R*- factors based on ALL data will be even larger.

### Fractional atomic coordinates and isotropic or equivalent isotropic displacement parameters (Å^2^)

**Table:**

	*x*	*y*	*z*	*U*_iso_*/*U*_eq_	
C1	0.7768 (6)	0.9797 (6)	0.1920 (4)	0.032 (2)	
H1	0.7820	0.9899	0.1489	0.038*	
C2	0.7942 (7)	1.0542 (7)	0.2377 (4)	0.042 (2)	
H2	0.8143	1.1232	0.2324	0.050*	
C3	0.7765 (6)	1.0081 (7)	0.2914 (4)	0.036 (2)	
H3	0.7809	1.0400	0.3312	0.043*	
C4	0.8923 (6)	0.6155 (6)	0.2699 (4)	0.036 (2)	
H4	0.9111	0.5689	0.2393	0.043*	
C5	0.9391 (7)	0.6182 (7)	0.3310 (4)	0.042 (3)	
H5	0.9958	0.5751	0.3498	0.051*	
C6	0.8889 (7)	0.6945 (7)	0.3598 (4)	0.041 (2)	
H6	0.9034	0.7144	0.4024	0.049*	
C7	0.4925 (7)	0.7002 (6)	0.2441 (4)	0.039 (2)	
H7	0.4547	0.6665	0.2088	0.047*	
C8	0.4520 (7)	0.7148 (7)	0.3005 (4)	0.045 (3)	
H8	0.3832	0.6945	0.3114	0.054*	
C9	0.5335 (7)	0.7650 (7)	0.3366 (4)	0.041 (2)	
H9	0.5305	0.7872	0.3783	0.050*	
C10	0.9845 (6)	0.7850 (6)	0.1509 (4)	0.030 (2)	
C11	1.0067 (6)	0.8209 (5)	0.2117 (4)	0.025 (2)	
H11	0.9496	0.8314	0.2365	0.030*	
C12	1.1155 (7)	0.8413 (6)	0.2360 (4)	0.032 (2)	
H12	1.1313	0.8671	0.2773	0.039*	
C13	1.1996 (7)	0.8246 (6)	0.2012 (4)	0.038 (2)	
H13	1.2728	0.8378	0.2185	0.046*	
C14	1.1762 (7)	0.7890 (7)	0.1416 (5)	0.045 (3)	
H14	1.2341	0.7772	0.1176	0.054*	
C15	1.0691 (7)	0.7694 (6)	0.1149 (4)	0.042 (3)	
H15	1.0541	0.7458	0.0730	0.050*	
C16	0.8175 (6)	0.8324 (6)	0.0499 (4)	0.029 (2)	
C17	0.8805 (7)	0.9189 (7)	0.0449 (4)	0.040 (2)	
H17	0.9409	0.9323	0.0757	0.048*	
C18	0.8558 (7)	0.9863 (7)	−0.0047 (4)	0.043 (3)	
H18	0.9011	1.0439	−0.0080	0.052*	
C19	0.7679 (8)	0.9711 (7)	−0.0486 (4)	0.044 (3)	
H19	0.7506	1.0186	−0.0816	0.053*	
C20	0.7051 (7)	0.8868 (7)	−0.0446 (4)	0.038 (2)	
H20	0.6445	0.8750	−0.0754	0.046*	
C21	0.7288 (6)	0.8174 (7)	0.0046 (3)	0.033 (2)	
H21	0.6837	0.7593	0.0069	0.040*	
C22	0.8747 (6)	0.6233 (6)	0.0854 (4)	0.034 (2)	
C23	0.8167 (7)	0.5810 (7)	0.0324 (4)	0.045 (3)	
H23	0.7608	0.6200	0.0091	0.054*	
C24	0.8378 (8)	0.4827 (7)	0.0121 (4)	0.052 (3)	
H24	0.7982	0.4568	−0.0252	0.062*	
C25	0.9146 (8)	0.4243 (7)	0.0455 (5)	0.051 (3)	
H25	0.9279	0.3571	0.0320	0.061*	
C26	0.9736 (7)	0.4623 (7)	0.0989 (4)	0.046 (3)	
H26	1.0280	0.4219	0.1223	0.055*	
C27	0.9530 (7)	0.5596 (7)	0.1183 (4)	0.037 (2)	
H27	0.9937	0.5846	0.1555	0.045*	
C28	0.5159 (7)	0.8916 (7)	0.1380 (4)	0.055 (3)	
H28A	0.4655	0.8603	0.1639	0.082*	
H28B	0.4744	0.9240	0.1015	0.082*	
H28C	0.5601	0.9430	0.1626	0.082*	
C29	0.6553 (8)	0.5105 (7)	0.1935 (4)	0.073 (3)	
H29A	0.7302	0.4939	0.2111	0.110*	
H29B	0.6223	0.4516	0.1705	0.110*	
H29C	0.6130	0.5278	0.2275	0.110*	
C30	0.3166 (9)	0.7761 (9)	−0.0453 (5)	0.103 (5)	
H30A	0.3553	0.7143	−0.0569	0.123*	
H30B	0.2924	0.7633	−0.0040	0.123*	
N1	0.7516 (5)	0.8910 (5)	0.2177 (3)	0.0273 (17)	
N2	0.7519 (5)	0.9104 (5)	0.2797 (3)	0.0305 (18)	
N3	0.8157 (5)	0.6886 (5)	0.2595 (3)	0.0272 (17)	
N4	0.8147 (5)	0.7359 (5)	0.3154 (3)	0.0284 (17)	
N5	0.5924 (5)	0.7400 (5)	0.2464 (3)	0.0326 (17)	
N6	0.6183 (5)	0.7785 (5)	0.3048 (3)	0.0299 (18)	
N7	0.5880 (5)	0.8123 (5)	0.1169 (3)	0.0316 (17)	
H7A	0.6220	0.8400	0.0854	0.038*	
H7B	0.5443	0.7603	0.0995	0.038*	
N8	0.6561 (5)	0.5967 (5)	0.1514 (3)	0.0396 (19)	
H8A	0.5859	0.6044	0.1320	0.047*	
H8B	0.6976	0.5780	0.1209	0.047*	
Cl1	0.4076 (2)	0.8772 (3)	−0.03824 (13)	0.0900 (10)	
Cl2	0.2051 (2)	0.7940 (2)	−0.09856 (13)	0.0811 (9)	
Cl3	0.40486 (19)	0.61848 (19)	0.08092 (10)	0.0552 (7)	
Ru1	0.71139 (5)	0.74571 (5)	0.18349 (3)	0.0266 (2)	
P1	0.84429 (16)	0.74734 (19)	0.11836 (9)	0.0289 (5)	
B1	0.7333 (7)	0.8233 (8)	0.3253 (5)	0.035 (3)	
H1C	0.7418	0.8472	0.3697	0.042*	
O1	0.4507 (5)	0.3851 (5)	0.0283 (3)	0.078 (2)	
H1A	0.4588	0.4381	0.0491	0.093*	
H1B	0.4911	0.3729	0.0012	0.093*	

### Atomic displacement parameters (Å^2^)

**Table:**

	*U*^11^	*U*^22^	*U*^33^	*U*^12^	*U*^13^	*U*^23^
C1	0.026 (5)	0.034 (6)	0.037 (6)	0.002 (4)	0.009 (4)	0.005 (5)
C2	0.045 (6)	0.033 (6)	0.048 (6)	−0.009 (5)	0.003 (5)	−0.004 (5)
C3	0.027 (5)	0.036 (6)	0.042 (6)	−0.002 (4)	−0.007 (4)	−0.014 (5)
C4	0.027 (5)	0.023 (5)	0.060 (7)	0.002 (4)	0.008 (5)	0.007 (5)
C5	0.023 (5)	0.049 (7)	0.052 (7)	−0.002 (5)	−0.006 (5)	0.021 (5)
C6	0.033 (6)	0.052 (7)	0.037 (6)	0.000 (5)	−0.001 (5)	0.013 (5)
C7	0.021 (5)	0.044 (6)	0.050 (6)	−0.011 (4)	−0.005 (5)	0.003 (5)
C8	0.024 (5)	0.074 (8)	0.038 (6)	−0.006 (5)	0.006 (5)	0.004 (5)
C9	0.034 (6)	0.061 (7)	0.030 (5)	0.005 (5)	0.007 (4)	0.007 (5)
C10	0.014 (5)	0.040 (6)	0.036 (5)	0.000 (4)	0.001 (4)	0.001 (4)
C11	0.026 (5)	0.018 (5)	0.031 (5)	0.004 (4)	0.006 (4)	0.003 (4)
C12	0.031 (6)	0.022 (5)	0.040 (6)	−0.014 (4)	−0.010 (5)	0.004 (4)
C13	0.014 (5)	0.040 (6)	0.058 (7)	−0.013 (4)	−0.005 (5)	−0.001 (5)
C14	0.022 (5)	0.051 (7)	0.066 (7)	−0.002 (5)	0.015 (5)	0.015 (5)
C15	0.028 (5)	0.056 (7)	0.042 (5)	−0.005 (5)	0.008 (4)	−0.014 (5)
C16	0.025 (5)	0.024 (5)	0.038 (5)	0.001 (4)	0.003 (4)	0.002 (4)
C17	0.032 (6)	0.040 (7)	0.048 (6)	0.008 (5)	0.010 (5)	−0.002 (5)
C18	0.040 (6)	0.040 (6)	0.053 (7)	−0.001 (5)	0.019 (5)	0.003 (5)
C19	0.042 (7)	0.038 (7)	0.056 (7)	0.002 (5)	0.019 (5)	0.013 (5)
C20	0.034 (6)	0.055 (7)	0.026 (5)	0.003 (5)	0.003 (4)	−0.008 (5)
C21	0.035 (6)	0.039 (6)	0.026 (5)	0.006 (4)	0.002 (4)	0.000 (4)
C22	0.029 (5)	0.033 (6)	0.041 (6)	−0.003 (4)	0.004 (4)	−0.009 (5)
C23	0.027 (6)	0.039 (7)	0.069 (7)	0.002 (5)	0.007 (5)	−0.014 (5)
C24	0.051 (7)	0.048 (7)	0.052 (7)	−0.008 (6)	−0.009 (5)	−0.019 (6)
C25	0.044 (7)	0.038 (7)	0.072 (8)	0.004 (5)	0.016 (6)	−0.010 (6)
C26	0.044 (6)	0.036 (6)	0.056 (7)	0.015 (5)	−0.002 (5)	0.002 (5)
C27	0.045 (6)	0.030 (6)	0.037 (6)	0.003 (5)	0.002 (4)	−0.007 (5)
C28	0.031 (6)	0.071 (8)	0.058 (7)	0.018 (5)	−0.010 (5)	0.013 (5)
C29	0.074 (8)	0.046 (7)	0.091 (8)	−0.009 (6)	−0.026 (6)	0.028 (6)
C30	0.062 (8)	0.119 (12)	0.122 (10)	−0.010 (8)	−0.007 (8)	0.067 (8)
N1	0.020 (4)	0.023 (4)	0.038 (5)	−0.005 (3)	0.000 (3)	0.008 (4)
N2	0.027 (4)	0.031 (5)	0.033 (5)	−0.004 (3)	0.001 (3)	−0.005 (4)
N3	0.023 (4)	0.029 (4)	0.029 (4)	−0.004 (3)	0.001 (3)	0.003 (3)
N4	0.026 (4)	0.028 (5)	0.029 (4)	−0.005 (3)	−0.002 (3)	0.006 (4)
N5	0.021 (4)	0.039 (5)	0.037 (4)	−0.002 (4)	0.002 (3)	0.010 (4)
N6	0.034 (5)	0.028 (5)	0.027 (4)	0.002 (3)	0.004 (3)	0.000 (3)
N7	0.020 (4)	0.034 (5)	0.040 (4)	0.004 (3)	0.000 (3)	0.002 (4)
N8	0.027 (4)	0.030 (5)	0.062 (5)	−0.001 (3)	0.010 (4)	0.009 (4)
Cl1	0.061 (2)	0.132 (3)	0.076 (2)	0.006 (2)	0.0063 (16)	−0.027 (2)
Cl2	0.080 (2)	0.075 (2)	0.084 (2)	−0.0087 (17)	−0.0043 (17)	0.0237 (17)
Cl3	0.0451 (16)	0.0703 (19)	0.0481 (16)	−0.0086 (14)	−0.0021 (12)	−0.0036 (13)
Ru1	0.0212 (4)	0.0249 (4)	0.0331 (4)	−0.0023 (4)	0.0009 (3)	0.0012 (4)
P1	0.0226 (12)	0.0289 (13)	0.0347 (13)	−0.0017 (12)	0.0025 (9)	−0.0029 (13)
B1	0.020 (6)	0.034 (7)	0.049 (7)	0.002 (5)	−0.001 (5)	0.000 (6)
O1	0.085 (5)	0.058 (5)	0.094 (6)	−0.004 (4)	0.028 (4)	0.005 (4)

### Geometric parameters (Å, º)

**Table:**

C1—N1	1.344 (9)	C21—H21	0.9500
C1—C2	1.385 (10)	C22—C23	1.387 (10)
C1—H1	0.9500	C22—C27	1.401 (10)
C2—C3	1.350 (10)	C22—P1	1.835 (9)
C2—H2	0.9500	C23—C24	1.397 (11)
C3—N2	1.335 (9)	C23—H23	0.9500
C3—H3	0.9500	C24—C25	1.355 (11)
C4—N3	1.348 (9)	C24—H24	0.9500
C4—C5	1.371 (10)	C25—C26	1.376 (11)
C4—H4	0.9500	C25—H25	0.9500
C5—C6	1.370 (11)	C26—C27	1.377 (10)
C5—H5	0.9500	C26—H26	0.9500
C6—N4	1.353 (8)	C27—H27	0.9500
C6—H6	0.9500	C28—N7	1.480 (9)
C7—N5	1.337 (9)	C28—H28A	0.9800
C7—C8	1.388 (10)	C28—H28B	0.9800
C7—H7	0.9500	C28—H28C	0.9800
C8—C9	1.363 (10)	C29—N8	1.452 (9)
C8—H8	0.9500	C29—H29A	0.9800
C9—N6	1.338 (9)	C29—H29B	0.9800
C9—H9	0.9500	C29—H29C	0.9800
C10—C11	1.387 (9)	C30—Cl2	1.695 (10)
C10—C15	1.397 (10)	C30—Cl1	1.735 (11)
C10—P1	1.854 (8)	C30—H30A	0.9900
C11—C12	1.406 (9)	C30—H30B	0.9900
C11—H11	0.9500	N1—N2	1.360 (8)
C12—C13	1.377 (10)	N1—Ru1	2.083 (6)
C12—H12	0.9500	N2—B1	1.545 (11)
C13—C14	1.364 (10)	N3—N4	1.359 (7)
C13—H13	0.9500	N3—Ru1	2.091 (6)
C14—C15	1.400 (10)	N4—B1	1.560 (11)
C14—H14	0.9500	N5—N6	1.357 (8)
C15—H15	0.9500	N5—Ru1	2.129 (6)
C16—C21	1.387 (9)	N6—B1	1.551 (10)
C16—C17	1.390 (10)	N7—Ru1	2.143 (5)
C16—P1	1.847 (8)	N7—H7A	0.9200
C17—C18	1.392 (10)	N7—H7B	0.9200
C17—H17	0.9500	N8—Ru1	2.156 (6)
C18—C19	1.362 (10)	N8—H8A	0.9200
C18—H18	0.9500	N8—H8B	0.9200
C19—C20	1.361 (11)	Ru1—P1	2.297 (2)
C19—H19	0.9500	B1—H1C	1.0000
C20—C21	1.400 (10)	O1—H1A	0.8276
C20—H20	0.9500	O1—H1B	0.8319
			
N1—C1—C2	110.0 (8)	C26—C27—H27	118.5
N1—C1—H1	125.0	C22—C27—H27	118.5
C2—C1—H1	125.0	N7—C28—H28A	109.5
C3—C2—C1	105.5 (9)	N7—C28—H28B	109.5
C3—C2—H2	127.3	H28A—C28—H28B	109.5
C1—C2—H2	127.3	N7—C28—H28C	109.5
N2—C3—C2	109.0 (8)	H28A—C28—H28C	109.5
N2—C3—H3	125.5	H28B—C28—H28C	109.5
C2—C3—H3	125.5	N8—C29—H29A	109.5
N3—C4—C5	110.1 (8)	N8—C29—H29B	109.5
N3—C4—H4	125.0	H29A—C29—H29B	109.5
C5—C4—H4	125.0	N8—C29—H29C	109.5
C6—C5—C4	107.1 (8)	H29A—C29—H29C	109.5
C6—C5—H5	126.4	H29B—C29—H29C	109.5
C4—C5—H5	126.4	Cl2—C30—Cl1	114.6 (6)
N4—C6—C5	106.3 (8)	Cl2—C30—H30A	108.6
N4—C6—H6	126.9	Cl1—C30—H30A	108.6
C5—C6—H6	126.9	Cl2—C30—H30B	108.6
N5—C7—C8	110.5 (8)	Cl1—C30—H30B	108.6
N5—C7—H7	124.7	H30A—C30—H30B	107.6
C8—C7—H7	124.7	C1—N1—N2	105.8 (7)
C9—C8—C7	104.1 (8)	C1—N1—Ru1	134.9 (6)
C9—C8—H8	128.0	N2—N1—Ru1	119.3 (5)
C7—C8—H8	128.0	C3—N2—N1	109.8 (7)
N6—C9—C8	110.1 (8)	C3—N2—B1	129.7 (8)
N6—C9—H9	125.0	N1—N2—B1	120.4 (7)
C8—C9—H9	125.0	C4—N3—N4	105.3 (6)
C11—C10—C15	120.3 (7)	C4—N3—Ru1	137.4 (6)
C11—C10—P1	120.7 (6)	N4—N3—Ru1	117.2 (5)
C15—C10—P1	118.8 (6)	C6—N4—N3	111.2 (7)
C10—C11—C12	118.7 (8)	C6—N4—B1	126.2 (7)
C10—C11—H11	120.7	N3—N4—B1	122.5 (6)
C12—C11—H11	120.7	C7—N5—N6	106.7 (7)
C13—C12—C11	121.5 (8)	C7—N5—Ru1	134.6 (6)
C13—C12—H12	119.3	N6—N5—Ru1	118.6 (5)
C11—C12—H12	119.3	C9—N6—N5	108.7 (7)
C14—C13—C12	118.9 (8)	C9—N6—B1	131.0 (7)
C14—C13—H13	120.5	N5—N6—B1	120.2 (7)
C12—C13—H13	120.5	C28—N7—Ru1	119.1 (5)
C13—C14—C15	121.8 (9)	C28—N7—H7A	107.5
C13—C14—H14	119.1	Ru1—N7—H7A	107.5
C15—C14—H14	119.1	C28—N7—H7B	107.5
C10—C15—C14	118.8 (8)	Ru1—N7—H7B	107.5
C10—C15—H15	120.6	H7A—N7—H7B	107.0
C14—C15—H15	120.6	C29—N8—Ru1	122.3 (5)
C21—C16—C17	117.5 (8)	C29—N8—H8A	106.8
C21—C16—P1	121.5 (6)	Ru1—N8—H8A	106.8
C17—C16—P1	120.8 (6)	C29—N8—H8B	106.8
C16—C17—C18	120.6 (8)	Ru1—N8—H8B	106.8
C16—C17—H17	119.7	H8A—N8—H8B	106.6
C18—C17—H17	119.7	N1—Ru1—N3	87.4 (2)
C19—C18—C17	121.2 (9)	N1—Ru1—N5	87.7 (3)
C19—C18—H18	119.4	N3—Ru1—N5	84.0 (2)
C17—C18—H18	119.4	N1—Ru1—N7	88.9 (2)
C20—C19—C18	119.1 (9)	N3—Ru1—N7	170.5 (2)
C20—C19—H19	120.5	N5—Ru1—N7	87.1 (2)
C18—C19—H19	120.5	N1—Ru1—N8	174.9 (2)
C19—C20—C21	120.8 (8)	N3—Ru1—N8	93.7 (2)
C19—C20—H20	119.6	N5—Ru1—N8	87.4 (3)
C21—C20—H20	119.6	N7—Ru1—N8	89.2 (2)
C16—C21—C20	120.7 (8)	N1—Ru1—P1	92.90 (19)
C16—C21—H21	119.6	N3—Ru1—P1	93.67 (19)
C20—C21—H21	119.6	N5—Ru1—P1	177.59 (17)
C23—C22—C27	115.4 (8)	N7—Ru1—P1	95.24 (18)
C23—C22—P1	124.4 (7)	N8—Ru1—P1	92.02 (18)
C27—C22—P1	119.8 (6)	C22—P1—C16	104.6 (4)
C22—C23—C24	122.0 (9)	C22—P1—C10	98.9 (4)
C22—C23—H23	119.0	C16—P1—C10	101.8 (4)
C24—C23—H23	119.0	C22—P1—Ru1	115.3 (3)
C25—C24—C23	120.2 (9)	C16—P1—Ru1	115.2 (3)
C25—C24—H24	119.9	C10—P1—Ru1	118.7 (3)
C23—C24—H24	119.9	N2—B1—N6	107.8 (6)
C24—C25—C26	120.0 (9)	N2—B1—N4	107.8 (7)
C24—C25—H25	120.0	N6—B1—N4	105.5 (7)
C26—C25—H25	120.0	N2—B1—H1C	111.8
C25—C26—C27	119.4 (8)	N6—B1—H1C	111.8
C25—C26—H26	120.3	N4—B1—H1C	111.8
C27—C26—H26	120.3	H1A—O1—H1B	120.1
C26—C27—C22	122.9 (8)		

### Hydrogen-bond geometry (Å, º)

**Table:**

*D*—H···*A*	*D*—H	H···*A*	*D*···*A*	*D*—H···*A*
N7—H7*B*···Cl3	0.92	2.54	3.430 (7)	164
N8—H8*A*···Cl3	0.92	2.37	3.293 (6)	178
O1—H1*A*···Cl3	0.83	2.58	3.342 (7)	154
O1—H1*B*···Cl3^i^	0.83	2.32	3.136 (7)	166

Symmetry code: (i) −*x*+1, −*y*+1, −*z*.
